# At-home testing to mitigate community transmission of SARS-CoV-2: protocol for a public health intervention with a nested prospective cohort study

**DOI:** 10.1186/s12889-021-12007-w

**Published:** 2021-12-04

**Authors:** Emily J. Ciccone, Donaldson F. Conserve, Gaurav Dave, Christoph P. Hornik, Marlena L. Kuhn, Jessica L. Herling, Michelle Song, Shani Alston, Lindsay Singler, Michael D. Schmidt, Aaron Jones, Samuel Broderick, Lisa M. Wruck, Warren A. Kibbe, Allison E. Aiello, Christopher W. Woods, Alan Richmond, Michael Cohen-Wolkowiez, Giselle Corbie-Smith

**Affiliations:** 1 Division of Infectious Diseases, University of North Carolina School of Medicine, Chapel Hill, NC USA; 2 Department of Prevention and Community Health, Milken Institute School of Public Health, George Washington University, Washington, DC USA; 3 Division of General Medicine and Clinical Epidemiology, University of North Carolina School of Medicine, Chapel Hill, NC USA; 4 Department of Pediatrics, Duke University School of Medicine, Durham, NC USA; 5 Center for Health Equity Research, Department of Social Medicine, University of North Carolina, Chapel Hill, NC USA; 6 Duke Clinical Research Institute, Duke University School of Medicine, Durham, NC USA; 7 Office of Technology, DataRobot, Inc, Boston, MA USA; 8 Department of Biostatistics and Bioinformatics, Duke University School of Medicine, Durham, NC USA; 9 Department of Epidemiology, Gillings School of Global Public Health, University of North Carolina, Chapel Hill, NC USA; 10 Departments of Medicine and Pathology, Duke University School of Medicine, Durham, NC USA; 11 Community-Campus Partnerships for Health, Raleigh, NC USA; 12 Center for Health Equity Research, Department of Social Medicine, Department of Medicine, University of North Carolina School of Medicine, Chapel Hill, NC USA

**Keywords:** COVID-19 pandemic, SARS-CoV-2 antigen testing, Public health initiative, Community engagement, Health behavior, Health equity, Non-pharmaceutical interventions

## Abstract

**Background:**

The COVID-19 pandemic caused by the severe acute respiratory syndrome coronavirus 2 (SARS-CoV-2) continues to evolve as a global health crisis. Although highly effective vaccines have been developed, non-pharmaceutical interventions remain critical to controlling disease transmission. One such intervention—rapid, at-home antigen self-testing—can ease the burden associated with facility-based testing programs and improve testing access in high-risk communities. However, its impact on SARS-CoV-2 community transmission has yet to be definitively evaluated, and the socio-behavioral aspects of testing in underserved populations remain unknown.

**Methods:**

As part of the Rapid Acceleration of Diagnostics–Underserved Populations (RADx-UP) program funded by the National Institutes of Health, we are implementing a public health intervention titled “Say Yes! COVID Test” (SYCT) involving at-home self-testing using a SARS-CoV-2 rapid antigen assay in North Carolina (Greenville, Pitt County) and Tennessee (Chattanooga City, Hamilton County). The intervention is supported by a multifaceted communication and community engagement strategy to ensure widespread awareness and uptake, particularly in marginalized communities. Participants receive test kits either through online orders or via local community distribution partners. To assess the impact of this intervention on SARS-CoV-2 transmission, we will conduct a non-randomized, ecological study using community-level outcomes. Specifically, we will evaluate trends in SARS-CoV-2 cases and hospitalizations, SARS-CoV-2 viral load in wastewater, and population mobility in each community before, during, and after the SYCT intervention. Individuals who choose to participate in SYCT will also have the option to enroll in an embedded prospective cohort substudy gathering participant-level data to evaluate behavioral determinants of at-home self-testing and socio-behavioral mechanisms of SARS-CoV-2 community transmission.

**Discussion:**

This is the first large-scale, public health intervention implementing rapid, at-home SARS-CoV-2 self-testing in the United States. The program consists of a novel combination of an at-home testing program, a broad communications and community engagement strategy, an ecological study to assess impact, and a research substudy of the behavioral aspects of testing. The findings from the SYCT project will provide insights into innovative methods to mitigate viral transmission, advance the science of public health communications and community engagement, and evaluate emerging, novel assessments of community transmission of disease.

**Supplementary Information:**

The online version contains supplementary material available at 10.1186/s12889-021-12007-w.

## Background

The coronavirus disease 2019 (COVID-19) pandemic continues to evolve as a global public health crisis. As of July 2021, 34 million people in the United States have been infected with the SARS-CoV-2 virus, and over 600,000 have died [1]. Infections and deaths have disproportionally affected historically marginalized populations [2–4]. Although multiple highly effective SARS-CoV-2 vaccines have been rapidly developed and are now available, public health measures remain critical to controlling the pandemic [5]. Vaccination rates vary widely by state, with vaccine accessibility, hesitancy, misinformation, and mistrust contributing to incomplete uptake, particularly among historically underrepresented groups [6–9]. Furthermore, the Centers for Disease Control and Prevention (CDC) has recorded more than 10,000 breakthrough infection cases, [10] contributing to vaccine hesitancy. The durability of response to the vaccines is currently unknown, with some of the most recent variants potentially impacting the effectiveness of the vaccines [11, 12]. Also, the effectiveness of the vaccines in certain groups, such as those who are immunocompromised, is still being assessed [13]. With the current reality of vaccination challenges, there continues to be an urgent need to develop novel, effective mitigation measures, such as non-pharmaceutical interventions (NPIs), that prioritize historically underrepresented populations [5].

The availability of rapid tests for detecting SARS-CoV-2 presents opportunities for self-administered, frequent at-home testing in asymptomatic populations as part of a broader NPI strategy, including masking, physical distancing, handwashing, contact tracing, and isolation. Early testing strategies suffered from limited access to testing sites and long wait times for results [14]. Periodic home testing can overcome these barriers by facilitating convenient testing, identifying SARS-CoV-2 index cases early, triggering isolation and quarantine precautions, and ultimately decreasing community transmission [15]. With 50% or more of infections resulting from presymptomatic or asymptomatic transmission, [16] frequent at-home testing may also offer a practical option for screening for and breaking transmission chains. However, previous at-home testing studies have generated inconclusive results due to inadequate community engagement strategies for promoting consistent testing in the target population and limited testing and prevention knowledge, attitudes, and behavior [17, 18]. A public health intervention of frequent at-home testing could ease the burden of large-scale facility-based testing programs, provide communities with an additional course of action to reduce the impact of COVID-19, and catalyze urgent action to address needs in high-risk communities. This type of initiative requires intentional engagement with the community and a clear recognition of the risk of SARS-CoV-2 infection to individuals and the community.

Building on our experience with engaging communities for health interventions, and in partnership with the National Institutes of Health (NIH) and the CDC, we launched a public health intervention titled “Say Yes! COVID Test” (SYCT) to determine whether rapid, at-home testing reduces SARS-CoV-2 community transmission and to explore human behavioral factors affecting viral testing in two counties in the southeastern United States that include underserved populations. This project consists of four components: the at-home testing public health intervention, a broad communication and community engagement strategy, an ecologic study of the effectiveness of the public health intervention, and a research substudy assessing behavioral aspects of home testing. Here we report the design, methods, and ecological analysis of SYCT. A more detailed description of communications and community engagement strategies will be included in future papers.

## Methods/design

### Partnerships

To implement and evaluate the community rollout of SYCT, we leveraged national partnerships in community engagement; clinical trials operations; informatics; data collection, integration, and dissemination; biostatistics; public health; implementation science; and engagement science (Table 1). We collaborated with the NIH, CDC, state and local health departments, and community, research, and corporate partners. We also capitalized on the existing robust infrastructure among the partnering research team, including the Rapid Acceleration of Diagnostics–Underserved Populations (RADx-UP) program, the University of North Carolina (UNC) Center for Health Equity Research, the Duke Clinical Research Institute, Community-Campus Partnerships for Health (CCPH), and North Carolina Central University.

**Table 1:** Say Yes! COVID Test (SYCT) program partners and collaborators and their respective roles

Institution	Role
NIH	Study funder; provided test kits, approved protocol, and implementation plans, led SYCT launch, and coordinated study activities among stakeholders
CDC	Assisted with protocol development and selection of SYCT communities, engaged local and state health departments
RADx-UP Coordination and Data Collection Center (Duke Clinical Research Institute and the UNC Center for Health Equity Research)	Led design of SYCT intervention and ecological analysis, consulted on public health intervention protocol development, implementation of community-based distribution; created, designed, and implemented marketing campaign for public health intervention; consulted on design and implementation of community engagement plan; tracked kit distribution from local distribution partners; performed data integration and analysis, and manuscript preparation; responsible for all elements of SYCT substudy design, survey development, data management and analysis, and reporting
NCCU	Assisted with design and implementation of community engagement strategy and identification of local distribution partners
CCPH	Led design and implementation of community engagement plan for SYCT; led engagement and relationship building efforts with local distribution partners
University of Massachusetts	Data management partner for the SYCT substudy
Pitt County (NC) Health Department	Coordinated test distribution and local distribution partner engagement for SYCT in Pitt County, NC
Chattanooga (TN) Health Department	Coordinated test distribution and local distribution partner engagement for SYCT in Chattanooga, TN
Quidel	Manufactured at-home tests; applied for and received emergency use authorization from FDA
CareEvo	Designed the study mobile application; coordinated online ordering portal and distribution of kits via Amazon
Amazon	Distributed test kits requested through an online ordering system
DataRobot	Conducted simulations during planning for the public health intervention to guide community selection
Noble Laboratory (UNC)	Processed and tested wastewater samples from North Carolina
Biobot Analytics, Inc.	Processed and tested wastewater samples from Tennessee

*Abbreviations: CCPH* Community-Campus Partnerships for Health, *CDC* Centers for Disease Control and Prevention, *FDA* US Food and Drug Administration, *NCCU* North Carolina Central University, *NIH* National Institutes of Health, *RADx-UP* Rapid Acceleration of Diagnostics–Underserved Populations, *UNC* University of North Carolina

### Study design

**Fig. 1:**
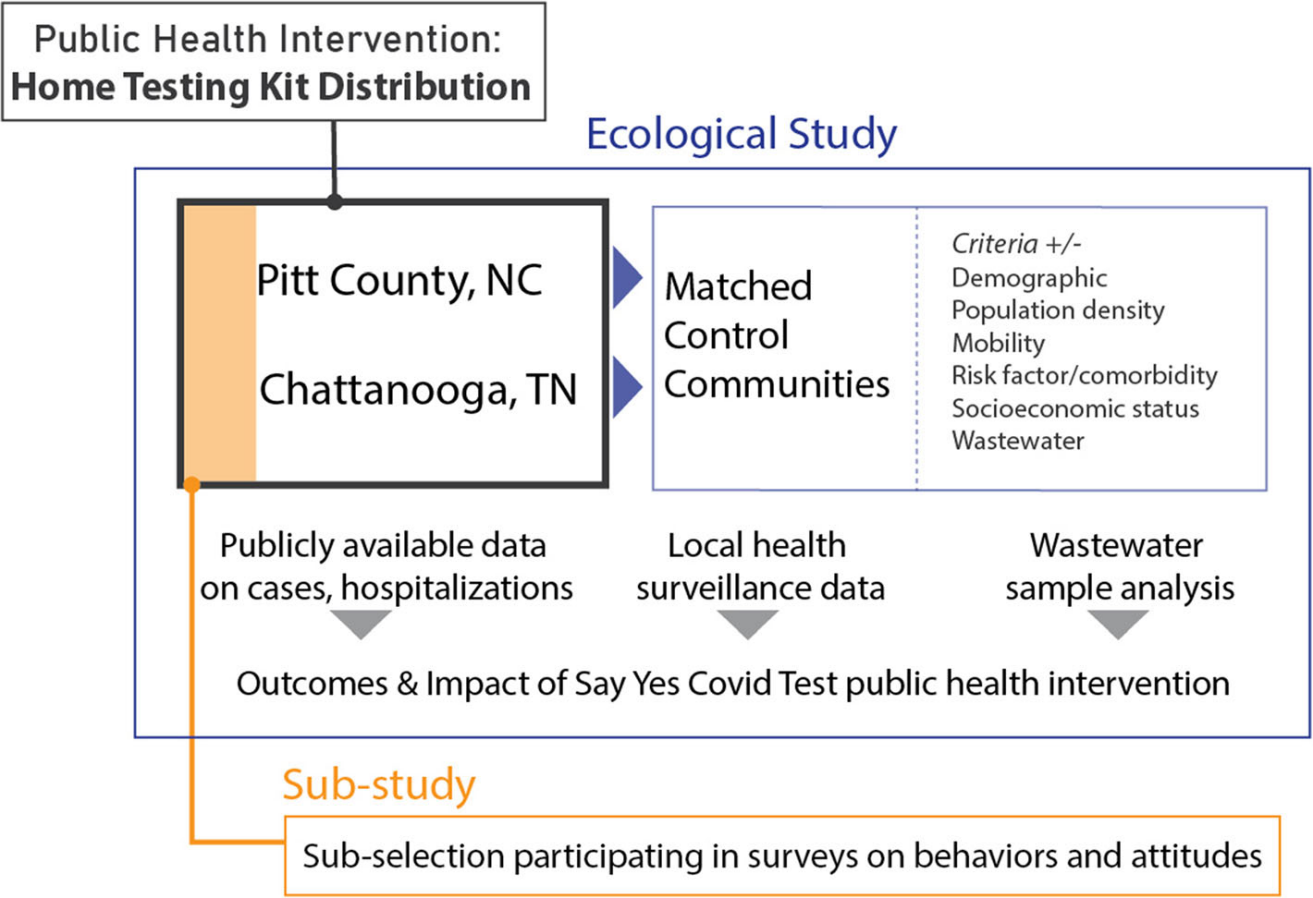
Schematic of Say Yes! COVID Test program components

**Fig. 2:**
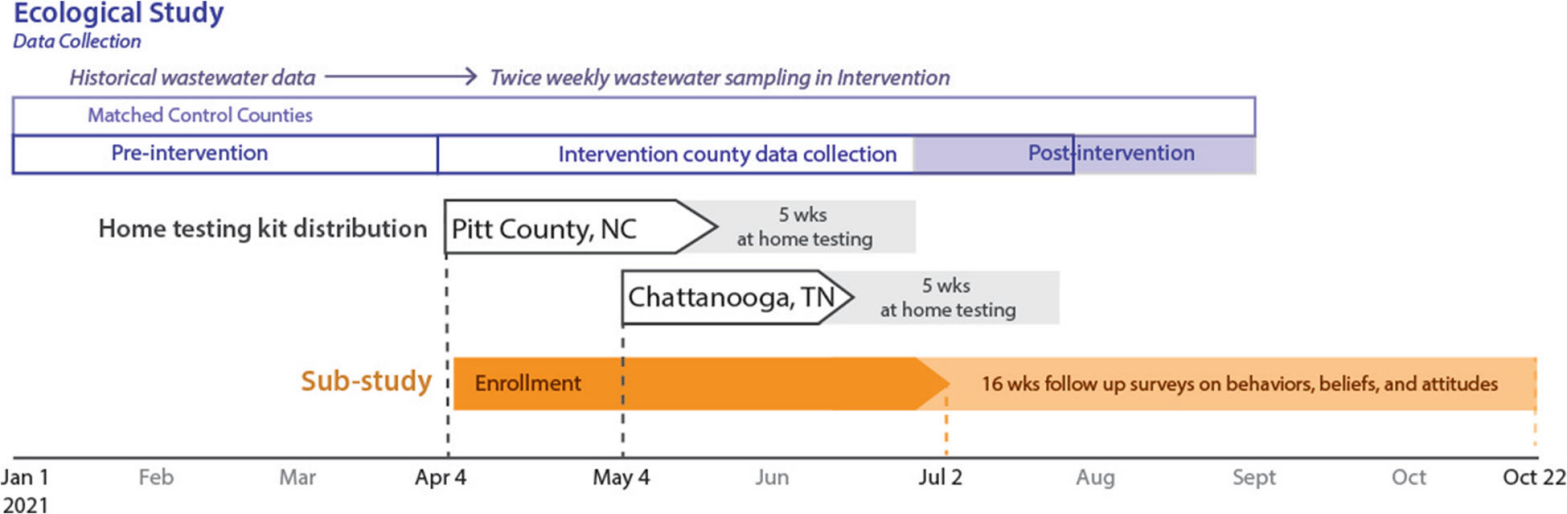
Timeline of Say Yes! COVID Test program activities and data collection

We are performing a non-randomized ecological analysis to assess the impact of the public health intervention consisting of at-home, self-administered SARS-CoV-2 testing (SYCT) on community transmission (Figs. 1 and 2). Specifically, we will use publicly reported SARS-CoV-2 testing, hospitalization, mortality, and vaccination data, augmented by detailed surveillance data from the health departments on community SARS-CoV-2 transmission. In addition, SARS-CoV-2 wastewater testing will be performed.

Individuals who chose to participate in the SYCT intervention could also enroll in the embedded prospective cohort substudy. The SYCT substudy focuses on gathering participant-level data to evaluate behavioral determinants of home testing and socio-behavioral mechanisms of SARS-CoV-2 community transmission (Fig. 1). We hypothesize that positive at-home test results will be associated with altered self-reported social interactions and altered health behaviors compared to negative test results. After obtaining informed consent, participants will complete periodic surveys and questionnaires through a smartphone application or call center phone calls. Questionnaires will collect data on demographic characteristics, medical history, health status, COVID testing, symptoms, social interactions, knowledge of prevention strategies, infection risk, and attitudes toward vaccines. We will also ask the participants of the substudy to report the results of their SARS-CoV-2 tests.

### Setting and population

#### Study sites

In identifying potential sites for the intervention, we worked with DataRobot, Inc. to conduct a modeling exercise to estimate the likely impact of frequent self-testing on SARS-CoV-2 prevalence, incidence, and hospitalizations in cities of various geographic sizes (see Additional file 1 for the complete list of key assumptions). We assessed the impact under the following testing protocol parameters:

1. All household members (based on the average household size per location) take tests uniformly over 4 weeks;
2. All household members (based on the average household size per location) take tests uniformly over 5 weeks;
3. Two people per household test every 2 days for 4 weeks;
4. Two people per household test every 3 days for 5 weeks;
5. Control case in which no additional household testing was conducted.

We considered numerous community-level factors in selecting intervention sites, controlling for age distribution in each location by adjusting the model for the severity and infection fatality ratio. Specifically, the inputs (and data sources) for the model included the following:

1. Incidence and prevalence of SARS-CoV-2 disease (Johns Hopkins University [JHU]);
2. Population size and density (US Census);
3. Immunity (due to SARS-CoV-2 disease and vaccine distribution anticipated at varied dates when intervention would be conducted);
4. Local SARS-CoV-2 reverse transcription polymerase chain reaction (PCR) testing (HHS Protect, US Department of Health & Human Services).

The DataRobot team identified 10 locations in California, Florida, Wisconsin, North Carolina, and Tennessee that optimized impact on critical outcomes, including percentage reduction in infections over the study period, the percentage reduction in incidence (i.e., new infections per capita on the final day of the study period), and percentage reduction in hospitalizations over the study period.

Following this exercise, we overlaid the results with the following information to identify preferable cities to conduct the intervention:

1. Availability of reliable, publicly available outcome data, including COVID-19 infections, hospitalizations, deaths, and vaccinations, as well as wastewater surveillance
2. Overlap with current RADx-UP sites to leverage the existing community-academic connections and infrastructure and to ensure inclusion of underserved populations;
3. Population size requirements to maximize the impact of 2 million tests available for the intervention across the selected sites.

**Fig. 3:**
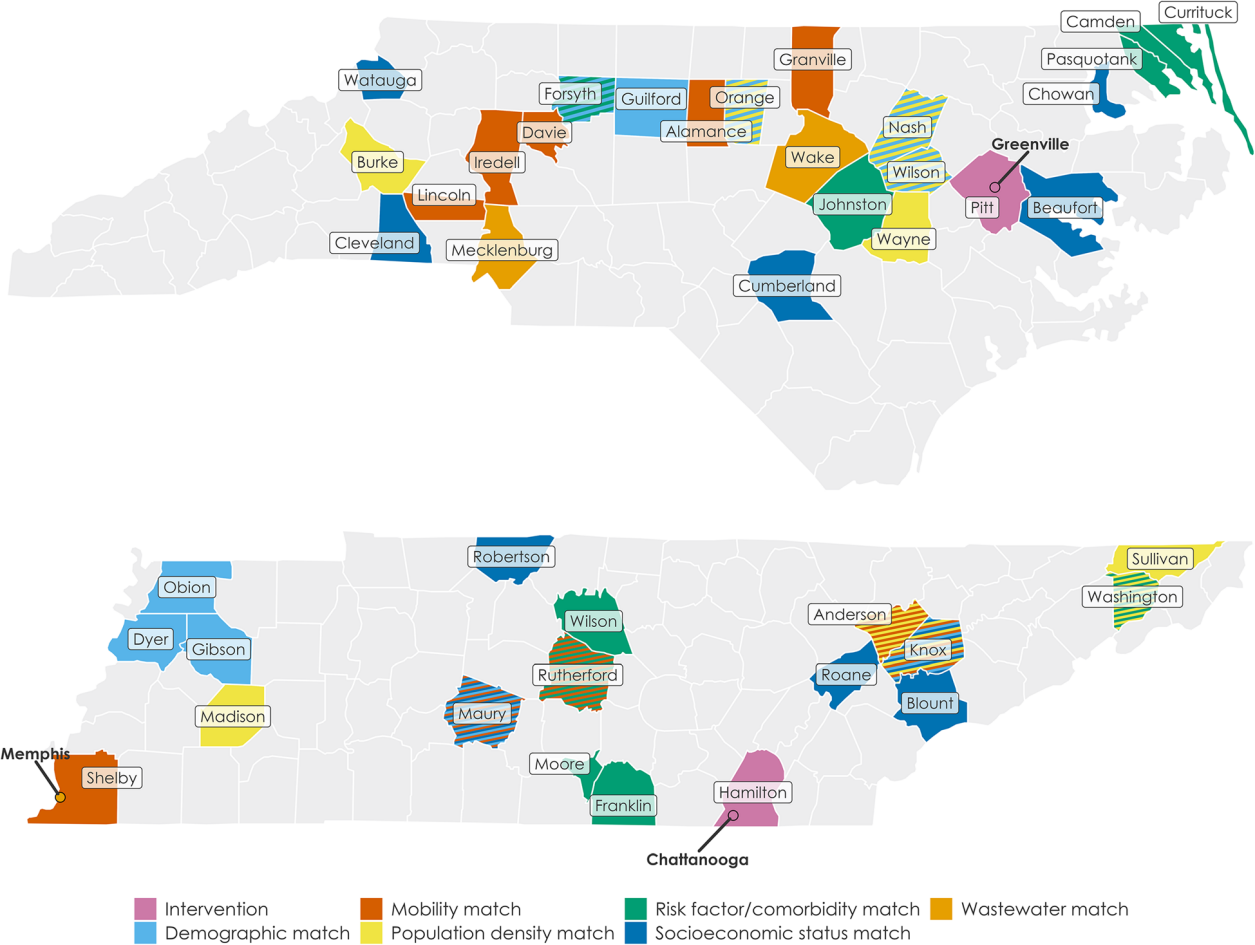
Map of Say Yes! COVID Test intervention counties and the five groups of matched controls communities for each state, comprising the best matches for each of the following categories of matching variables: demographics (including age distribution and racial and ethnic diversity), mobility, population density, risk factors/comorbidities, socioeconomic status (including per capita income), and incidence/vaccination trajectory

In consultation with state and local health departments, we selected the city of Greenville and Pitt County, NC, and the city of Chattanooga and Hamilton County, TN (40,000 test kits per site, each containing 25 tests per kit; Fig. 3) as the first sites for SYCT. We will be expanding the SYCT intervention and data collection for the ecological study to two additional communities by Fall 2021.

We also selected multiple matched control communities for each intervention community within the same state to control state-level trends and policies **(**Fig. 3**)**. Given the limited number of counties in each state and potential confounders, no single community or composite of communities was likely to be identified as a good match in every factor affecting the outcomes. Instead, we selected five groups of matched controls for each state, comprising the best matches for each of the following categories of matching variables: demographics (including age distribution and racial and ethnic diversity), mobility, population density, risk factors/comorbidities, socioeconomic status (including per capita income), and incidence/vaccination trajectory. We chose most of the matching categories and individual variables to mirror as much as possible the National Institute of Environmental Health Sciences COVID-19 Pandemic Vulnerability Index [19]. To select control communities for the wastewater analyses, we used wastewater data availability, comparability of the sewer shed, and consistency of lab procedures as criteria.

### SYCT intervention and ecological analysis

#### Study population

The study population comprises the residents of Pitt County (encompassing Greenville), North Carolina; Chattanooga City, Hamilton County, Tennessee; and matched control communities. Community members were encouraged to participate in the SYCT intervention if they had a self-reported primary residence within one of the pre-identified communities. Individuals under 8 years of age could not participate per the at-home test indications (see below).

#### SARS-CoV-2 test

**Fig. 4:**
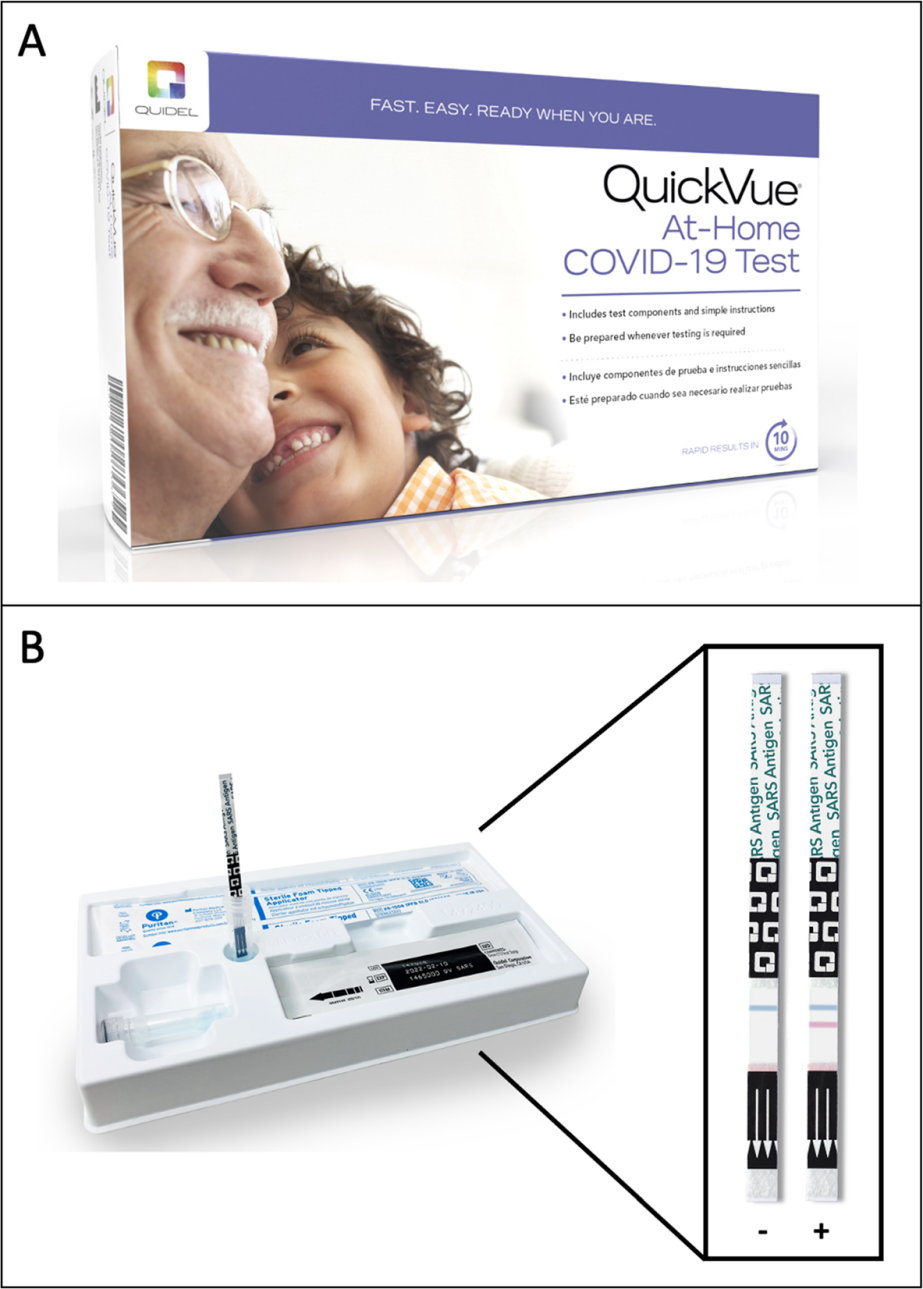
The at-home SARS-CoV-2 antigen test used for the Say Yes! COVID Test intervention, the Quidel QuickVue At-home COVID-19 Test (Panel A). Each kit came with 25 individual tests with results shown on a lateral flow test strip. The blue line represents the control line, whereas the red line appears only if the test is positive (Panel B)

The test used for the SYCT intervention was the QuickVue SARS-CoV-2 rapid antigen assay (Quidel Corporation, San Diego, CA; Fig. 4). The test received emergency use authorization for over-the-counter distribution and at-home use by individuals aged 8 years and older from the FDA in March 2021. Individuals aged 8–14 must have an adult present to perform the test. It identifies SARS-CoV-2 nucleocapsid protein, which is generally detectable in anterior nasal specimens during the acute phase of infection. Negative tests do not definitively rule out SARS-CoV-2 infection. To conduct the test, individuals self-collect an anterior nasal swab, mix the swab in a provided tube prefilled with the testing solution, and then place a test strip into the test solution. After 10 min, the strip gives either a positive or negative result. The test strip includes an internal control – if that line is not present, the test is considered invalid (see Additional file 2). Each test kit contains supplies to perform 25 individual tests.

#### Test distribution

**Fig. 5:**
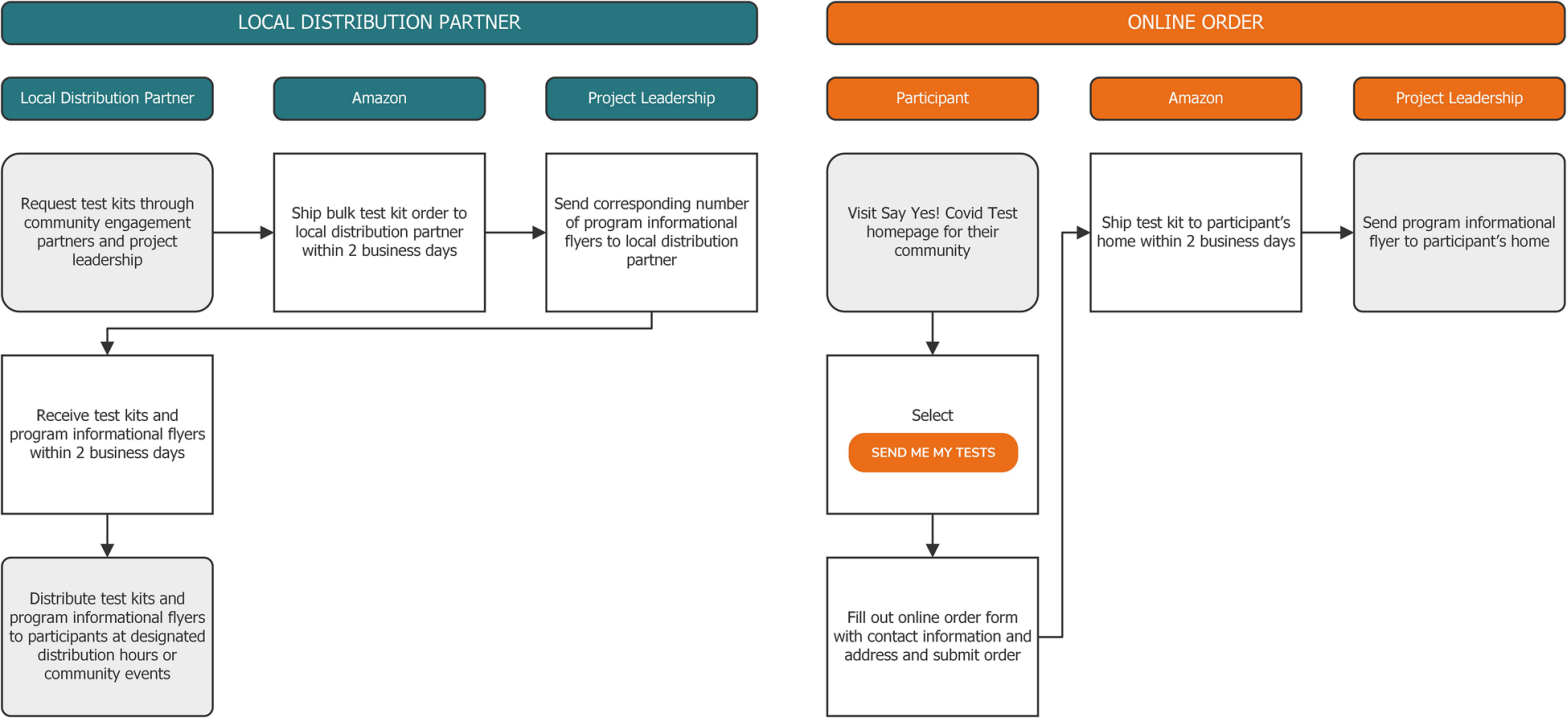
Visual representation of the two methods for distribution of Say Yes! COVID Test program test kits

Test kit distribution began in Pitt County on April 4, 2021, and Hamilton County on May 4, 2021. All test kits were stored in Amazon warehouses located near each community. Participants could receive test kits in two ways: online orders or local distribution partners **(**Fig. 5**).**

##### Online order distribution:

Participants accessed the ordering website for their community through the project’s homepage, www.sayyescovidtest.org, and entered their household address information. Each order was restricted to one test kit per address. To augment participation, starting in April, individuals placing an online order could also order an extra kit for a friend. The test kits were shipped via 2-day Amazon shipping directly to the participant’s house in an Amazon box. Concurrently, the program mailed out a flyer to their address to remind them about the program’s expectations. We timed the flyer to arrive at the same time as the test kit.

##### Community distribution:

The community engagement team and project leadership formed partnerships with local distribution partners within each participating community. The local health department also served as a local distribution partner. Each distribution partner requested several test kits for distribution via the community engagement team and project leadership. These requests were sent to Amazon for fulfillment as a bulk order. Amazon shipped the bulk order test kits to the local distribution partner via 2-day shipping. Project leadership provided each local distribution partner with a corresponding number of program information flyers. Local distribution partners then held events to give away the test kits or had designated pickup times at their center or business. We listed these events and pickup times on the community’s website for the program. Participants did not have to provide any contact information. Each participant received a program information flyer at the same time as test kit pickup with instructions on how to use the test kit to participate in the at-home testing initiative.

#### Community engagement strategy

Recruitment of key stakeholders and community leaders was led by the CCPH. The team leveraged an extensive network of contacts to quickly stand up an ad-hoc advisory group of community leaders in each SYCT county to develop a community-informed strategy for distribution of at-home test kits. A series of informal listening sessions were held with the initial community advisory groups to hear their views about community interest in piloting at-home testing for SARS-CoV-2, recommendations for distribution sites, and potential barriers for rapid community-wide distribution of the at-home test kits. CCPH staff also held a half-day strategic planning session to design a comprehensive plan for disseminating information about the availability of the test kits and process for organizing community partners to serve as distribution volunteers. Upon approval of distribution plan, a site manager and staff were hired in both communities to lead recruitment and training of volunteers and promotion of the SYCT campaign.

#### Communications and micromarketing campaign

We led a public health communications and micro-marketing campaign to build widespread awareness for SYCT across the two selected communities and encourage test kit use. We first named and branded the program with local community members’ input and developed key messages and supporting materials to promote the at-home testing intervention. We established a program website and social media channels, including YouTube, Facebook, and Instagram, to drive program awareness and facilitate test kit orders and use. We also developed and disseminated various print materials, including fliers, posters, bus shelter advertisements, door hangers, and billboards, as well as television and radio ads along with online ads. Community partners received branded facemasks, promotional materials, feather flags, and distribution tents to promote local test kit distribution events. Finally, we led a public relations effort to engage national and local media outlets in sharing news of the program and information on how to participate. This strategy resulted in widespread coverage across print and digital media outlets, including CBS and NBC News features. The communications and micromarketing campaign ran through the 6-week distribution to facilitate test kit orders and continued for several weeks to remind participants to test three times a week until they used all test kits. Lessons learned in the use of communications and micromarketing to encourage awareness and uptake of at-home SARS-CoV-2 rapid tests in underserved populations will be fully described in a separate paper.

#### Testing protocol

**Fig. 6:**
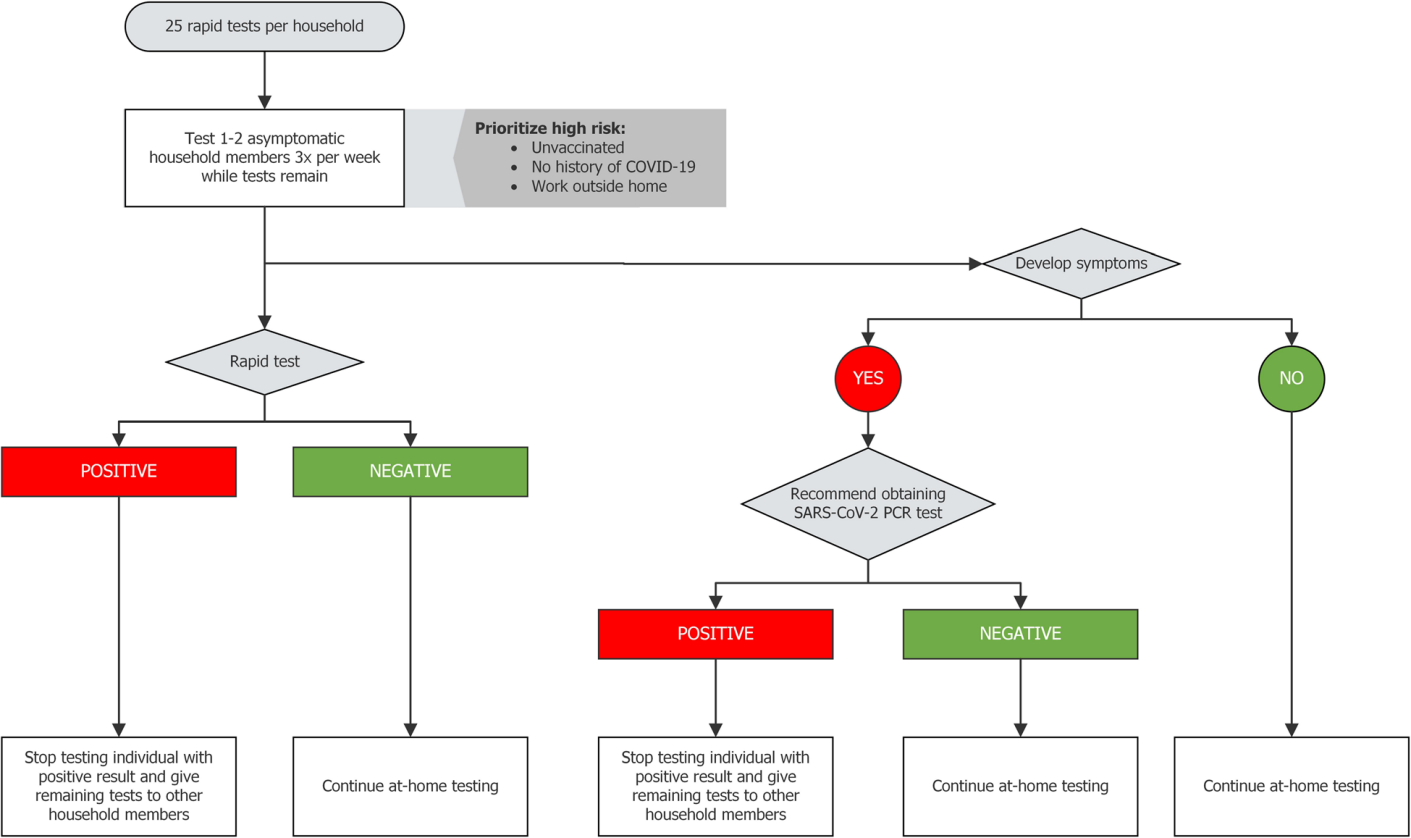
At-home testing protocol for Say Yes! COVID Test intervention

Each participating household received one kit of 25 tests each. All test kits included an informational brochure with clear written instructions with visual aids and links to videos in English and Spanish. We instructed the participants to test one or two asymptomatic individuals within the household three times per week until all tests were used (4–5 weeks; Fig. 6). We encouraged participants to prioritize household members with the highest risk of exposure to or infection with SARS-CoV-2, such as those who worked outside the home, were not vaccinated for SARS-CoV-2, or had not been previously infected. If an individual tested positive by rapid test, we instructed them to stop testing and give their remaining tests to another household member. The SYCT program did not require positive antigen test results reporting, although, for TN participants, a phone number was available for voluntary reporting.

If a participant developed any symptoms consistent with COVID-19 disease (e.g., fever, cough, shortness of breath), we recommended that the participant contact their primary physician or the local health department to obtain a PCR test for SARS-CoV-2. Reporting positive results to the health department was done through the testing institution; SYCT did not mandate result reporting. At the time of symptoms, we did not recommend additional at-home testing outside of the thrice-weekly schedule.

#### Participant support

The at-home testing kits and the marketing campaign included information about a study smartphone application and a Quick Response (QR) code to facilitate access. Developed by CareEvolution, the free app (called MyDataHelps) ran on Apple and Android devices [20]. Serving as a digital study resource, it included information about SYCT, instructions to administer at-home tests for all participants in the public health intervention, and information on how to enroll in the substudy.

#### Statistical considerations and data analysis

##### Outcomes and measures:

The data used to evaluate the impact of SYCT on participating communities will be publicly available, community-level data. The primary outcome is the number of positive SARS-CoV-2 tests per the local health department. The secondary outcomes fall into one of three categories: wastewater (e.g., SARS-CoV-2 viral copies per liter), mobility (e.g., percentage change in requests for driving directions), and other (e.g., hospitalizations attributable to SARS-CoV-2) (Table 2). We will also consider the following covariates: vaccination coverage, mobility data, and percentage of cases attributed to the B1.1.7 variant.

**Table 2:** Summary of primary and secondary outcomes for the ecological study of the SYCT program

Outcome Measures	Endpoints	Data source	Unit of observation	Frequency of data collection	Maximum geographic granularity
Primary					
SARS-CoV-2 test results	Community prevalence point estimate and 95% confidence interval; community incidence rate	Local health departments	County	Daily	County
Secondary					
SARS-CoV-2 viral copies per liter	Community transmission	Wastewater	Wastewater treatment plant sewer shed	Twice weekly	Wastewater treatment plant sewer shed
Number of new hospitalizations for COVID-19 disease per day	Hospitalizations	Local health departments	County	Daily	Center
Hospital census	Hospital capacity/Health care utilization	Local health departments	County	Daily	County/ Healthcare center
GPS location and length of stay; requests for driving directions; requests for walking directions	Mobility	Mobile phones (Google and Apple Maps)	County	Daily	City
SARS-CoV-2-positive patients admitted to the ICU	Number of ICU admissions	Local health department	Individual	Daily	Health care Center

*Abbreviation: ICU* Intensive Care Unit

##### Data collection procedures:

We will determine the daily case counts for ***community SARS-CoV-2 data*** using the JHU Coronavirus Resource Center for each selected county [1]. The daily case count data are made publicly available by the COVID-19 Data Repository operated by the Center for Systems Science and Engineering (CSSE) at JHU [21]. Documentation of the data by JHU is available at https://github.com/CSSEGISandData/COVID-19. This website contains a complete list of all sources used in the COVID-19 case data since January 21, 2020. There are 122 data sources for the county-level daily COVID-19 case count data. These data will be obtained through the ‘sars2pack’ R package (https://seandavi.github.io/sars2pack/) created by Sean Davis at the University of Colorado-Anschutz and VJ Carey. The case tracking data used for this study will include the date, count of cases, and the state and county to which the cases belong. We will obtain incidence data temporarily from the JHU source until the data resources on incident cases and other outcomes from the state health departments are successfully established.

The Google ***mobility data*** will be used to examine workplace, retail, grocery, and residential mobility. We defined workplace, retail, and grocery mobility as the number of visits to locations of each type; residential mobility is the duration of time spent at residential locations. The daily percentage change in mobility for each type of outcome is relative to a pre-pandemic baseline from early 2020 and is reported in Google’s COVID-19 Community Mobility Reports [22]. The 7-day average is computed from the daily percentage change for a selected county within a state. Among matched control communities during the study period beginning January 1, 2021, the daily percentage change is missing for approximately 3–11% of county-date pairs for each type of mobility. Excluding communities with > 50% missing values for a given category, the missing proportions are approximately 0–4% for each type of mobility. In these cases, the statewide average change for that day is used to impute the missing data. All mobility outcome values are fully observed for both intervention communities. The pre-pandemic baseline is a set of days representing the normal number of visits to workplace locations for a given day of the week. Therefore, the baseline is made up of seven individual values and is not a single value. The baseline for a particular day of the week is the median value from the 5 weeks from January 3 to February 6, 2020. The daily percentage change for a given day of the week is calculated by comparing the number of visits for the given day with the baseline for that given day.

The Apple mobility outcomes include the number of the driving route and walking route requests on Apple Maps. The daily percentage change in the number of a given type of request (driving or walking) is relative to the number of requests for that type made on January 13, 2020. We will obtain these data from Apple’s Mobility Trends Reports. The data and their documentation are available at https://covid19.apple.com/mobility.

The outcome variable for ***wastewater surveillance*** is normalized SARS CoV-2 concentrations, expressed as viral copies per liter. Twice weekly, 24-h composite wastewater samples are obtained at each sampling location, following the testing laboratory’s recommendations. For the Tennessee sites, the samples are taken according to the protocol and kits provided by Biobot Analytics, Inc., and tested by Biobot [23]; testing began on April 26, 2021. The North Carolina sites are being sampled and assayed under a contract through the CDC National Wastewater Surveillance System, with all the samples processed and assayed through the laboratory of Dr. Rachel Noble at UNC-Chapel Hill; sample collection is ongoing and started in September 2020. Samples are drawn from all locations on Monday and Thursday. The collection follows a standard 24-h cumulative sampling methodology. Historical biweekly samples and analyses are available from June 2020 through April 2021 for Chattanooga, weekly samples and analyses are available from October 2020 through mid-February 2021 for the MC Stiles facility in Memphis, and twice-weekly samples and analyses for the North Carolina sites will be available from September 2020 through April 2021. For the public health intervention, continuous twice-weekly sampling through the end of August will occur for North Carolina, with a 3-month follow-up after the intervention period. Continuous twice-weekly sampling through the end of September will occur for Tennessee. Analyte testing in North Carolina and Tennessee is performed using qPCR. The analysis reports from Biobot performed on samples collected in the Moccasin Bend Wastewater Treatment Plant in Chattanooga, TN, are publicly accessible at https://connect.chattanooga.gov/covid-biobot-analysis-reports/. These reports are generated and sent to the customer within 48 h of the receipt of the sample. In the analysis, the SARS-CoV-2 viral concentrations are calculated, normalized using internal controls, rainfall, the population covered by the sewer shed, and wastewater flow data.

##### Sample size and power calculation:

We did not perform a formal power calculation since this is an ecological study. We anticipate that approximately 40,000 households in each intervention community (NC and TN) will participate based on test kit availability. Due to the limited number of intervention communities and only observing outcomes at the community level, there is insufficient power to conduct formal hypothesis tests about the effect of the intervention. Therefore, the proposed analysis is strictly descriptive rather than inferential.

##### Analysis plan:

We will conduct analyses using SAS 9.4 (SAS Institute, Cary NC) or R 4.0 with R Studio 1.4 (R-project). Community characteristic summaries will be reported for each community, and specific variables included will be based on American Community Survey data. Categorical variables will be presented as estimates of counts and percentages with their corresponding 90% confidence intervals, and continuous variables as point estimates of the mean with their corresponding 90% confidence intervals. We will also present trends in community policies directed at pandemic control.

We will plot our outcomes of interest over time stratified by the community, delineating the pre-intervention, intervention, and post-intervention periods. Pre-intervention is defined as from January 1, 2021, until the start date of test kit distribution in each community. The total intervention period is approximately 11–12 weeks, defined as the 6-to-7-week period in which test kits are distributed with an additional 4-week period in which testing is implemented within households, plus a 1-week window to capture household delays usage of the kits. The post-intervention period includes the 90 days after the end of the intervention period. Daily outcomes will have 7-day rolling averages plotted over time at each daily time point. The results from wastewater samples are reported twice each week. The results from those samples will be plotted over time with 7-day rolling averages computed from all tests in that window. The plots of outcomes over time will be stratified by each community within a state.

We will use mixed modeling techniques to compare trends over time between intervention and control communities. Specifically, for each outcome and intervention/control community matched pair, a generalized linear model of 7-day average or twice-weekly outcomes by community and time with a time-by-community interaction will be fit, adjusted for vaccination coverage and the percentage of SARS-CoV-2 cases attributable to the B1.1.7 variant. Correlated residuals will be modeled within a community with an autoregressive correlation structure. The community will be modeled with a categorical fixed effect. Time will be modeled using restricted cubic splines. Four knots will be considered: at the beginning, at the transition from one period to the next, and at the end. Piecewise linear splines by period will also be considered for adequate fit. Linear time-by-community interactions will be included for each time. Vaccination coverage and percent of SARS-CoV-2 cases attributable to the B1.1.7 variant (estimated from wastewater data) will be modeled as a time-varying covariate where available, transformed as needed. The appropriate distribution and respective canonical link function will be used for each outcome type on the multiplicative/log scale when possible—i.e., binomial if a proportion, Poisson if discrete, log-transformed, and normal if continuous. Each intervention community will be compared to each matched control community separately within the intervention periods. This will be done by comparing the appropriate time-by-community interaction parameters using a likelihood ratio test with alpha = 0.05. This analysis will be repeated for the wastewater viral concentrations data.

Trends in mobility outcomes should be interpreted with caution. The outcome will reflect trends on the day of the week not corrected by the baseline value. The analytic team will consider smoothing methods to mitigate this effect.

### Behavioral substudy

#### Study population

Adults and children > 8 years of age living within the two communities currently participating in the public health intervention were eligible for the substudy.

#### Study questionnaires

Those who provide consent and enroll in the substudy are asked to complete questionnaires through an app and report any positive test results. Participants complete surveys and questionnaires through the smartphone app or via the call center according to the schedule of events (Additional file 3)**.** Questionnaires collect data on demographic characteristics, medical history, and health status; SARS-CoV-2 at-home testing and symptoms and any additional PCR testing obtained in response to symptoms; social interactions; knowledge of prevention strategies; infection risk; and attitudes toward vaccines. Questionnaires are completed during the week after each test (regardless of the result). We did not track participant-reported deviations to the recommended SYCT testing protocol.

#### Questionnaire distribution

The MyDataHelps smartphone application developed by CareEvolution (see ‘SYCT Intervention and Ecological Analysis’ section above) is also being used for the SYCT behavioral substudy [20]. Once registered, if the participant elects to be a part of the substudy, eligibility criteria are verified, and e-consent is obtained through the app. Push notifications are programmed with text messaging to promote adherence, including personalization, context, and timing for all participants in the public health intervention. Additionally, participants can opt-in to reminders for testing three times a week at a time of their preference. For participants who provided consent to the substudy, the app also features the ability to report their test results (upload images of test trips), track testing history, respond to surveys and questionnaires, and access the study team’s contact information.

Alternatively, participants who choose not to use the app can provide their information via phone interviews conducted by a centralized study call center. Using scripted interview guides, call center staff members explain study participation, obtain verbal consent, administer study questionnaires including soliciting test results, and issue phone reminders.

#### Statistical considerations and data analysis

##### Outcomes and measures:

The substudy hypothesis is that positive at-home test results will be associated with altered self-reported social interactions and altered health behaviors compared to negative test results. Our outcomes of interest for the substudy are the behavioral determinants of testing (social interaction, health behaviors, healthcare utilization, prevention knowledge, and vaccine attitudes). All outcome measures will be self-reported through the study questionnaires on an individual level. The frequency of measurement is described in Additional file 3**.** Outcome measures and endpoints are outlined in Table 3**.**

**Table 3:** Primary and secondary outcome measures and endpoints for the SYCT substudy

Outcome Measures	Endpoint
**Primary**	
Self-reported data on social distancing, quarantine, social connectedness, healthcare utilization, well-being	The proportion of respondents who report adhering to social distancing guidelines after a test result for the entire study cohort and stratified by participant demographics (e.g., gender) of interest; comparison of proportion adherent after positive vs. negative test result
**Secondary**	
Self-reported data on awareness of the issue, engagement, decisions to act, action, and maintenance	The proportion of respondents who decide to act on precautionary behaviors after a test result for the entire study cohort and stratified by participant demographics (e.g., gender) of interest
Self-reported emergency department visits, hospitalizations, and intensive care unit admission, for SARS CoV-2 evaluation or treatment	Point estimate and 95% confidence interval for each healthcare utilization measure and composite measure for the entire study cohort and stratified by participant demographics (e.g., gender) of interest
Self-reported data on perceptions and prevention of risks of contracting SARS CoV-2 infection	The proportion of respondents who are knowledgeable of precautionary measures to prevent infection for the entire study cohort and stratified by participant demographics (e.g., gender) of interest
Reported results of self-administered SARS CoV-2 antigen test	Prevalence of positive test results with 95% confidence intervals for the entire study cohort and stratified by participant demographic variables of interest
**Exploratory**	
Self-reported data on acceptability, practicality, integration, penetration, and demand of at-home self-administered antigen test	The proportion of respondents who find at-home self-administered antigen test acceptable for the entire study cohort and stratified by participant demographics (e.g., gender) of interest
Mobility patterns as captured by surrogates, including activity trackers	Distribution of individual mobility data in response to positive vs. negative SARS CoV-2 test results
Self-reported data on perceived susceptibility, severity, benefits, barriers, and cues to action related to SARS CoV-2 vaccination	Distribution of respondent’s perception about SARS CoV-2 vaccination for the entire study cohort and stratified by participant demographics (e.g., gender) of interest

##### Sample size and power calculation:

We did not perform a formal power calculation for this observational study. Instead, we anticipated sample size of 5000 participants or 6–7% of the expected 80,000 participants in the public health intervention who take up at-home testing. This sample size will be sufficient to estimate positive test incidence and prevalence, describe behavioral changes associated with test results, and conduct multivariable modeling to understand the causal chain between frequent testing and community burden of disease. For example, if the proportion of participants who adhere to social distancing is 50%, our sample size will be sufficient at a confidence level of 95% to estimate the prevalence with a margin of error of 1.5%.

##### Data analysis plan:

The proposed analysis is primarily descriptive rather than inferential without a priori planned formal hypothesis testing. We designed it to provide a general assessment of participant-level behaviors in a community testing intervention context. An exploratory hypothesis is that positive at-home SARS-CoV-2 antigen test results will lead to altered self-reported social interactions and altered health behaviors compared to negative test results. Demographics, questionnaire data, and testing results will be reviewed and summarized using graphical techniques and summary statistics. Where applicable, we will compute exact method confidence intervals around point estimates, and plot trends over time graphically. We will explore the associations between positive test results and critical behavioral measures using mixed modeling techniques to account for within-participant and within household correlations. SAS 9.4 (SAS Institute, Cary NC) or R 4.0 with R Studio 1.4 (R-project) will be used for statistical analyses.

## Discussion

The SYCT program assesses the impact and behavioral framework of a novel mitigation strategy—rapid, at-home, SARS-CoV-2 testing—implemented through a direct-to-consumer, community-engaged approach that minimizes participant burden and accelerates the translation of test results into daily life. The innovation of this initiative lies in its design, which bypasses the traditional research and healthcare environments and instead brings testing and study procedures to the participants’ homes. This approach minimizes risks by leveraging community engagement and intervention dissemination efforts and maximizes rewards by selectively complementing ecological study data with participant-specific information on behaviors and clinical outcomes associated with frequent at-home testing. In addition, we include both high-tech and low-tech pathways for participants to broaden the accessibility of the intervention and the research study to groups that are traditionally underrepresented.

Implementation and analysis of the SYCT program present significant challenges. The need for rapid deployment of an at-home testing intervention, given the ever-changing and urgent nature of the pandemic, requires the leveraging of local and national resources to employ a broad and multifaceted community engagement strategy. In addition, the use of an ecological study design to assess the impact of the SYCT intervention at the community level limits our ability to conduct formal hypothesis testing; instead, the analysis will be primarily descriptive as opposed to inferential. However, we will maximize our ability to document SYCT’s effect by evaluating an innovative combination of multiple community-level outcomes, using emerging scientific techniques such as wastewater sampling and testing, and analyzing large mobility data sources. In addition, we will be implementing the SYCT program and associated ecological study in an additional two communities before the end of 2021.

Even with the expanding uptake of vaccination, non-pharmaceutical interventions will remain critical components of pandemic control [24]. Therefore, easily accessible, effective testing strategies must be rigorously studied to inform public health policy. The SYCT program will provide critically needed insights into innovative methods to mitigate viral transmission, advance the science of community engagement, and evaluate emerging, novel assessments of community transmission of disease. Our unique, multifaceted approach will allow us to answer questions regarding community transmission for the SARS-CoV-2 pandemic and the socio-behavioral framework of at-home testing relevant to implementing public health interventions for both the current and future pandemics.

## Supplementary Information

**Additional file 1.** Modeling of Estimated Impact of SARS-CoV-2 Self-testing. This figure provides a detailed description of the key assumptions included in the modeling of the estimated impact of at-home SARS-CoV-2 rapid antigen testing on SARS-CoV-2 transmission in candidate communities.

**Additional file 2.** QuickVue At-Home COVID-19 Test: User Instructions. The instructions for use of the at-home rapid antigen test that were included with the test kits. Reproduced with permission of Quidel Corporation.

**Additional file 3.** Summary of SYCT substudy activities. This table includes timing and content of questionnaires.

## Data Availability

Not applicable.

## Abbreviations

CCPH: Community-Campus Partnerships for Health
CDC: Centers for Disease Control and Prevention
COVID-19: coronavirus disease 2019
NIH: National Institutes of Health
NPIs: non-pharmaceutical interventions
RADx-UP: Rapid Acceleration of Diagnostics–Underserved Populations
SARS-CoV-2: severe acute respiratory syndrome coronavirus 2
SYCT: “Say Yes! COVID Test”
