# Correction to: At-home testing to mitigate community transmission of SARS-CoV-2: protocol for a public health intervention with a nested prospective cohort study

**DOI:** 10.1186/s12889-021-12442-9

**Published:** 2022-01-21

**Authors:** Emily J. Ciccone, Donaldson F. Conserve, Gaurav Dave, Christoph P. Hornik, Marlena L. Kuhn, Jessica L. Herling, Michelle Song, Shani Alston, Lindsay Singler, Michael D. Schmidt, Aaron Jones, Samuel Broderick, Lisa M. Wruck, Warren A. Kibbe, Allison E. Aiello, Christopher W. Woods, Alan Richmond, Michael Cohen-Wolkowiez, Giselle Corbie-Smith

**Affiliations:** 1 Division of Infectious Diseases, University of North Carolina School of Medicine, Chapel Hill, NC USA; 2 Department of Prevention and Community Health, Milken Institute School of Public Health, George Washington University, Washington, DC USA; 3 Division of General Medicine and Clinical Epidemiology, University of North Carolina School of Medicine, Chapel Hill, NC USA; 4 Department of Pediatrics, Duke University School of Medicine, Durham, NC USA; 5 Department of Social Medicine, Center for Health Equity Research, University of North Carolina, Chapel Hill, NC USA; 6 Duke Clinical Research Institute, Duke University School of Medicine, Durham, NC USA; 7 Office of Technology, DataRobot, Inc., Boston, MA USA; 8 Department of Biostatistics and Bioinformatics, Duke University School of Medicine, Durham, NC USA; 9 Department of Epidemiology, Gillings School of Global Public Health, University of North Carolina, Chapel Hill, NC USA; 10 Departments of Medicine and Pathology, Duke University School of Medicine, Durham, NC USA; 11 Community-Campus Partnerships for Health, Raleigh, NC USA; 12 Department of Social Medicine, Center for Health Equity Research, University of North Carolina School of Medicine, Chapel Hill, NC USA

**Correction to BMC Public Health 21, 2209 (2021)**

**https://doi.org/10.1186/s12889-021-12007-w**

After publication of the original article [1] the authors noticed 2 errors in the acknowledgement section. The incorrect and correct acknowledgement section are published below, the changes are shown in **bold.**

**Incorrect acknowledgement**

- We acknowledge the immense contribution of all the community members who participated in the SYCT intervention and substudy, as well as the community partners (including local health departments) who worked with the SYCT program to encourage participation and facilitate kit distribution. Additionally, we thank Rachael Fleurence, the Office of the Director, and Dr. Michael Lauer from the NIH for their leadership and drive, as well as Elizabeth DiNenno from the CDC for her advice on study design and interfacing with health departments. Finally, we would like to acknowledge Jared Shamwell, Peter Fox, and Oleg Baranovskiy from DataRobot for their contributions to the site selection simulations; Charice Tellez and Jeannine Mason from Quidel for providing the images used in Fig. 4; CareEvolution for their mobile application support; Dr. Michael Muhammad with CCPH for his assistance with drafting the Community Engagement section of the manuscript; and Bola Yusuf for her organization of the manuscript preparation meetings and assistance with citations.

**Correct acknowledgement**

- We acknowledge the immense contribution of all the community members who participated in the SYCT intervention and substudy, as well as the community partners (including local health departments) who worked with the SYCT program to encourage participation and facilitate kit distribution. Additionally, we thank **Dr**. Rachael Fleurence, the Office of the Director, and Dr. Michael Lauer from the NIH for their leadership and drive, as well as **Dr**. Elizabeth DiNenno from the CDC for her advice on study design and interfacing with health departments. Finally, we would like to acknowledge **Dr**. Jared Shamwell, Peter Fox, and Oleg Baranovskiy from DataRobot for their contributions to the site selection simulations; Charice Tellez and Jeannine Mason from Quidel for providing the images used in Fig. 4; CareEvolution for their mobile application support; Dr. Michael Muhammad with CCPH for his assistance with drafting the Community Engagement section of the manuscript; **Dr. Claire Duvallet at Biobot for her assistance with the design and interpretation of the wastewater analysis**; and Bola Yusuf for her organization of the manuscript preparation meetings and assistance with citations.

The original article has been updated with the correct information.
