# Supplementary material for: At-home testing to mitigate community transmission of SARS-CoV-2: protocol for a public health intervention with a nested prospective cohort study
Source: BMC Public Health. 2021 Dec 4;21:2209. doi: 10.1186/s12889-021-12007-w (PMC8642753; doi:10.1186/s12889-021-12007-w)
Supplement: Supplementary file 1 — Additional file 1. Modeling of Estimated Impact of SARS-CoV-2 Self-testing. This figure provides a detailed description of the key assumptions included in the modeling of the estimated impact of at-home SARS-CoV-2 rapid antigen testing on SARS-CoV-2 transmission in candidate communities. [file 12889_2021_12007_MOESM1_ESM.pdf]

Overview of Key Assumptions for Modeling of Estimated Impact of SARS-CoV-2 Self-testing (1/2)

Assumption	Value
Study Period	February 1 to March 7 (5 weeks)
Test taking period	4 or 5 weeks depending on scenario (see previous page)
Test kit size	1 kit of of 25 tests per household
Test Cost	$10 per test
Testing Frequency	Varies by scenario (see previous page)
Maximum participants per location	50% of the population
Reduction in interaction from positive at-home test (RCF)*	Sampled between 50% and 90%
Test device sensitivity	95% sensitive (constant sensitivity starting at 10^6 copies / mL)
% of tests taken correctly*	Sampled between 63% and 95%
Combined overall test sensitivity*	Varies between 60% and 90%
Contagiousness over time	(see next page)
Asymptomatic Rate	30% weighted average across age groups
% of participants that take the test regularly throughout study	40% (discussed range 30 to 50%)

Combined test sensitivity =
(device sensitivity) x (% of tests taken correctly)

Legend

New from previous analysis
Varied assumption ranges

** For uncertainty analysis, we assume that the actual studies will fall within the parameter ranges shown with 95% probability.*

DataRobot **Confidential.** ©2020 DataRobot, Inc. – All rights reserved

Overview of Key Assumptions for Modeling of Estimated Impact of SARS-CoV-2 Self-testing (2/2)

Viral load over time by infection severity path

We assume viral load of an infected person varies by the severity of the infection (rows) and by day of infection (columns). We use the following viral load by infection severity path assumptions in our model.

Viral load per severity over time, copies/ml															
	Day 1	Day 2	Day 3	Day 4	Day 5	Day 6	Day 7	Day 8	Day 9	Day 10	Day 11	Day 12	Day 13	Day 14	Day 15
**Path 0R**	0.0E+00	1.0E+05	9.5E+05	2.9E+06	2.8E+07	2.8E+07	2.8E+07	2.9E+06	3.1E+05	1.0E+05	5.1E+04	5.1E+04	5.1E+04	5.1E+04	0.0E+00
**Path 1R**	0.0E+00	1.0E+05	9.5E+05	2.9E+06	4.3E+07	2.6E+08	8.5E+07	2.9E+06	3.1E+05	1.0E+05	5.1E+04	5.1E+04	5.1E+04	5.1E+04	0.0E+00
**Path 2R**	0.0E+00	1.0E+05	9.5E+05	2.9E+06	6.8E+07	4.1E+08	2.6E+08	2.9E+06	9.5E+05	1.0E+05	5.1E+04	5.1E+04	5.1E+04	5.1E+04	0.0E+00
**Path 3R**	0.0E+00	1.0E+05	9.5E+05	2.9E+06	1.1E+08	6.5E+08	4.1E+08	2.9E+06	9.5E+05	1.0E+05	5.1E+04	5.1E+04	5.1E+04	5.1E+04	0.0E+00
**Path 3D**	0.0E+00	1.0E+05	9.5E+05	2.9E+06	1.7E+08	2.5E+09	6.5E+08	2.9E+06	9.5E+05	1.0E+05	5.1E+04	5.1E+04	5.1E+04	5.1E+04	0.0E+00

Sources: Kucirka et al., 2020; Pujada et al., 2020 Scola et al., 2020

Infection severity progression paths

Each infection follows a specific severity path with different viral load

0R: Asymptomatic → Recovered
1R: Pre-Symp → Mild → Recovered
2R: Pre-Symp → Mild → Severe → Recovered
3R: Pre-Symp → Mild → Severe → Critical → Severe → Recovered
3D: Pre-Symp → Mild → Severe → Critical → Death

Corresponding device sensitivity by viral load

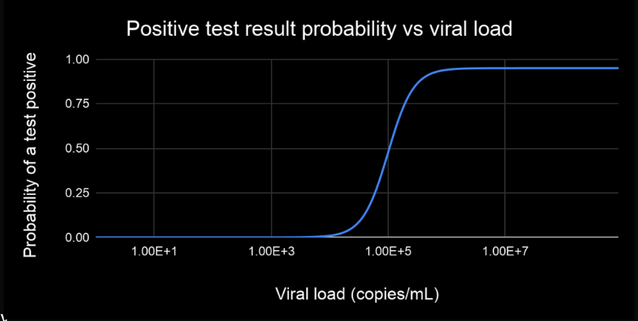

Infection path stages have durations with some stochasticity.

DataRobot Confidential. ©2020 DataRobot, Inc. – All rights reserved
