# Supplementary material for: At-home testing to mitigate community transmission of SARS-CoV-2: protocol for a public health intervention with a nested prospective cohort study
Source: BMC Public Health. 2021 Dec 4;21:2209. doi: 10.1186/s12889-021-12007-w (PMC8642753; doi:10.1186/s12889-021-12007-w)
Supplement: Supplementary file 2 — Additional file 2. QuickVue At-Home COVID-19 Test: User Instructions. The instructions for use of the at-home rapid antigen test that were included with the test kits. Reproduced with permission of Quidel Corporation. [file 12889_2021_12007_MOESM2_ESM.pdf]

QuickVue® At-Home COVID-19 Test

QUIDEL®

USER INSTRUCTIONS

For Emergency Use Authorization (EUA) only.
In vitro diagnostic use only. Rx only.

Visit safercovid.org/mytest or scan the code to the right to access these instructions, along with other key information, on your mobile phone.

Keep out of the reach of children. Contains hazardous chemicals.

Wash Your Hands

Before you start testing, wash your hands or use hand sanitizer.

Make sure your hands are dry before starting.

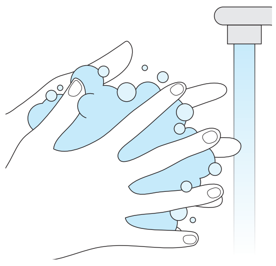

STEP 1
Check Your Test Kit

Blow your nose with a tissue and wash your hands or use hand sanitizer. Remove and identify the 5 parts of your test kit.

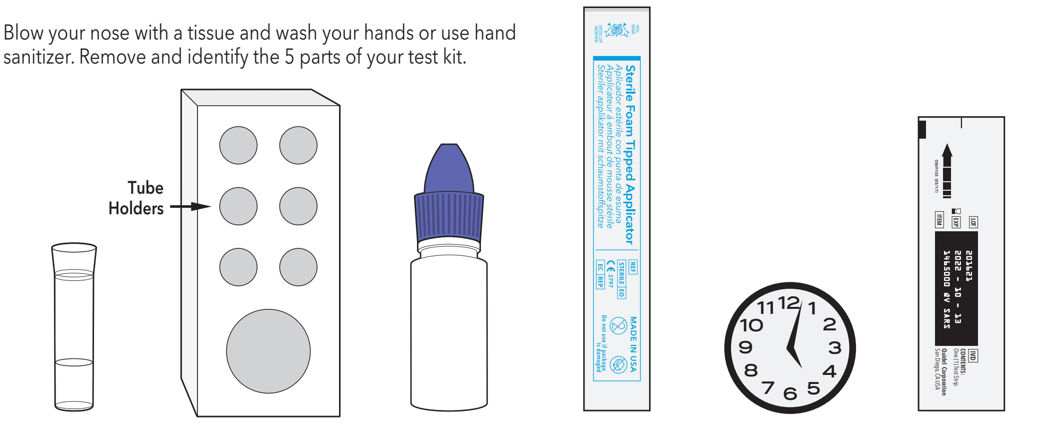

Twenty-five (25) Tubes · One (1) Kit Box · One (1) Bottle of Solution · Twenty-five (25) Swabs · Watch or Timer (not included) · Twenty-five (25) Strips

STEP 2
Dispense Solution

Uncap the **Bottle of Solution** and dispense the **Solution** up to the fill line on the **Tube** (approximately 7 drops).

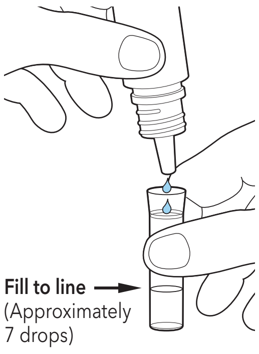

STEP 3
Secure Tube

Place the **Tube** in the **Tube Holder** on the **Kit Box**.

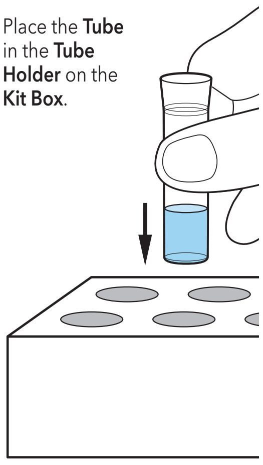

STEP 4
Swab the Nostrils

A Remove the **SWAB** from its wrapper, being careful not to touch the **SWAB** head.

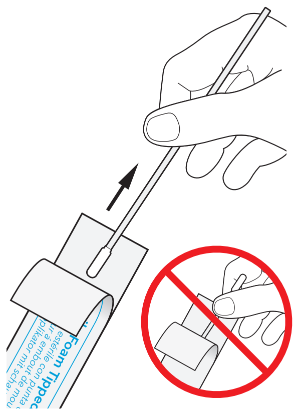

B Gently insert the **SWAB** ½ to ¾ of an inch into the nostril, depending on the size of the person's nose. Firmly rub the **SWAB** in a circular motion around the inside wall of **EACH NOSTRIL at least 4 times**.

Be sure to rub BOTH nostrils with the SAME SWAB.

NOTE: If you are swabbing others, please wear a face mask. With children, you may not need to insert the swab as far into the nostril. For very young children, you may need another person to steady the child's head while swabbing.

NOTE: Failure to swab properly may cause false negative results.

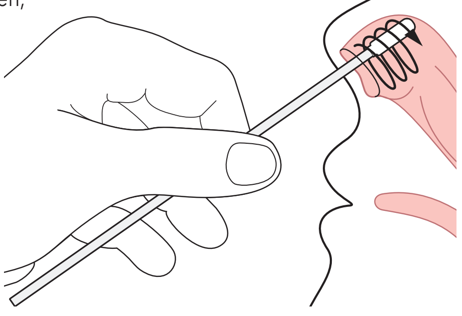

STEP 5
Place Swab in the Tube

A Immediately place the **SWAB** into the liquid inside the **TUBE**, and ensure it is touching the bottom. Stir 3-4 times.

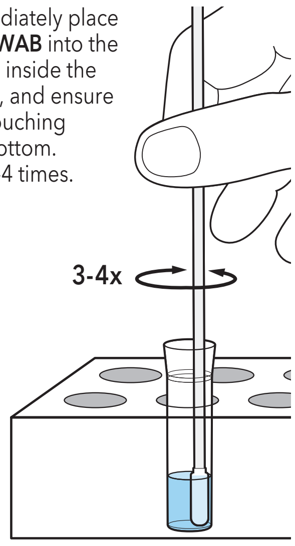

B Leave the swab in the solution for **ONE MINUTE**.

NOTE: If the swab is in the solution for more than 10-minutes it should not be used.

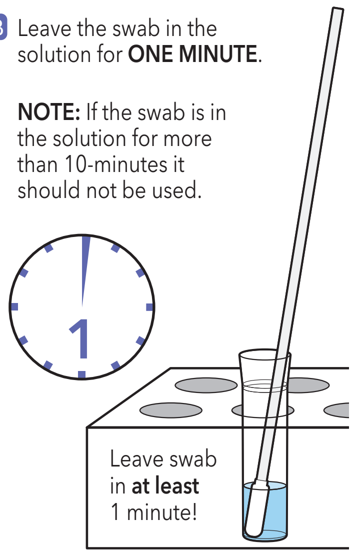

STEP 6
Remove Swab from the Tube

A After **ONE MINUTE**, remove the swab from the **TUBE** by rubbing the swab head against the inside wall of the tube to squeeze out as much liquid as possible.

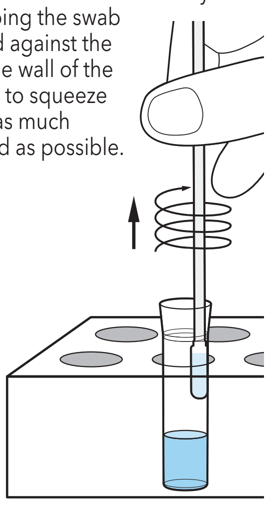

B Dispose of swab in the trash.

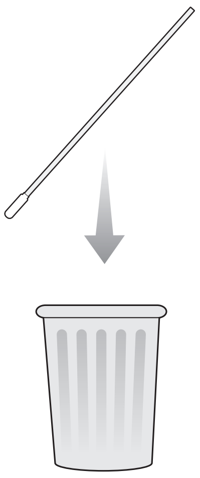

STEP 7
Open the Test Strip

Open the **TEST STRIP** pouch carefully at the slit and hold the **TEST STRIP** as indicated.

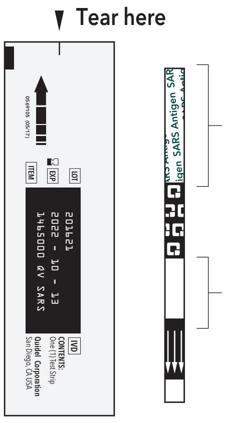

STEP 8
Place Test Strip in the Tube

Place the **TEST STRIP** into the **TUBE** with the arrows pointing down. Leave the strip in the **TUBE** for a **FULL TEN MINUTES** – do not handle or remove.

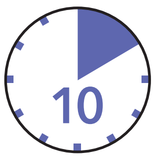

Leave strip in **at least** 10 minutes!

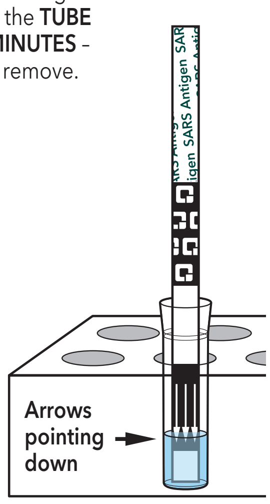

STEP 9
Remove Test Strip from the Tube

At **TEN MINUTES**, remove the **TEST STRIP** from the **TUBE** and place on the test strip outline in Steps 11-13. Ensure the **TEST STRIP** is on a flat surface in good lighting.

NOTE: The test is intended to be read at 10 minutes. If the test is read before this or is read more than 5 minutes after the indicated read time, results may be inaccurate (false negative, false positive, or invalid) and the test should be repeated.

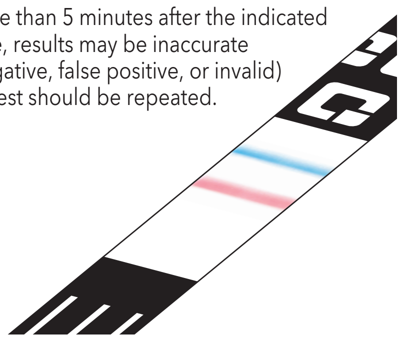

STEP 10
Check Your Results

There are three type of results possible.

1. Check for a Positive Result
2. Check for a Negative Result
3. Check for an Invalid Result

STEP 11
Check for a Positive COVID-19 Result

Place the **TEST STRIP** on the test strip outline below and compare with test result examples shown.

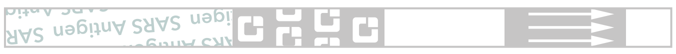

Positive Result

A **POSITIVE** result must show **BOTH** a **BLUE** line and a **PINK** line near the **BLUE** line.

Look closely! Even a very faint, pink Test Line and a blue Control Line is a POSITIVE result. The intensity of the lines may vary.

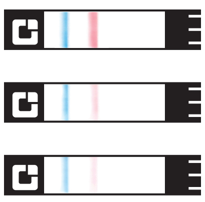

Results shown at 2x.

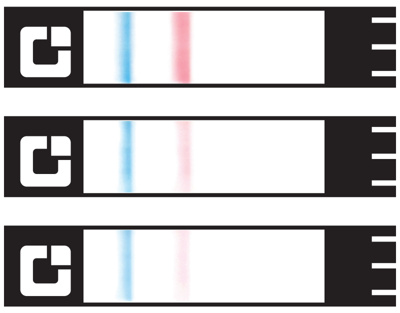

Positive COVID-19 Result

A positive test result means that proteins from the virus that causes COVID-19 were found in your sample and it is very likely you have COVID-19 and it is important to be under the care of your healthcare provider. It is also likely that you may be placed in isolation to avoid spreading the virus to others. There is a very small chance that this test can give you a positive test result that is wrong (false positive.) If you test positive with the QuickVue At-Home COVID-19 Test you should self-isolate and seek follow-up care with your healthcare provider as additional testing may be necessary. Your healthcare provider will work with you to determine how best to care for you based on your test result along with your medical history, and your symptoms.

Continued on other side

QuickVue® At-Home COVID-19 Test

QUIDEL®

SAY YES! COVID TEST
JOIN THE FREE AT-HOME TESTING CHALLENGE

Continued from other side

Keep out of the reach of children. Contains hazardous chemicals.

STEP 12

Check for a Negative COVID-19 Result

Place the **TEST STRIP** on the test strip outline below and compare with test result examples shown.

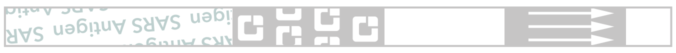

Negative Result

A **NEGATIVE** result will show a **BLUE** line but **NO PINK** line.

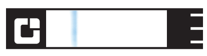

Results shown at 2x.

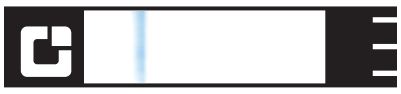

Negative COVID-19 Result

A negative test result means that proteins from the virus that causes COVID-19 were not found in your sample.

It is possible for this test to give a negative result that is incorrect (false negative) in some people with COVID-19. This means that you could possibly still have COVID-19 even though the test is negative. If you test negative and continue to experience COVID-19 like symptoms of fever, cough and/or shortness of breath you should seek follow up care with your healthcare provider. Your healthcare provider will consider the test result together with all other aspects of your medical history (such as symptoms, possible exposures, and geographical location of places you have recently traveled) in deciding how to care for you. For example, your healthcare provider may suggest you need another test to determine if you have contracted the virus causing COVID-19.

It is important that you work with your healthcare provider to help you understand the next steps you should take.

STEP 13

Check for an Invalid COVID-19 Result

Place the **TEST STRIP** on the test strip outline below and compare with test result examples shown.

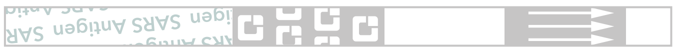

Invalid Result

If there is **NO LINE**, or if there is **ONLY** a **PINK** line, the test is **INVALID** and you should repeat the steps starting at the beginning using a new **TUBE**, **SWAB**, and **TEST STRIP**.

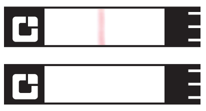

Results shown at 2x.

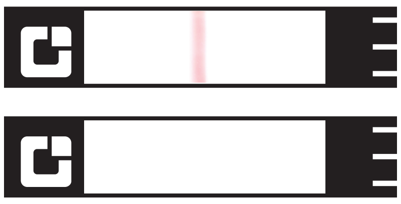

Invalid COVID-19 Result

If at 10 minutes, the blue Control Line does not appear, even if any shade of pink-to-red Test Line appears, the result is invalid. If the Test Result is invalid, a new swab should be collected, and the test should be performed again with a new pre-filled tube and Test Strip.

If the second QuickVue At-Home COVID-19 Test is also INVALID, call 833-QUICKVUE (833-784-2588) for assistance.

STEP 14

Dispose Used Test in the Trash

All used test components should be disposed of in your household waste.

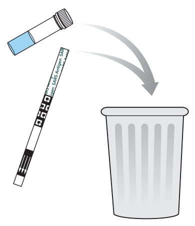

Wash Your Hands

After completing all steps, wash hands or use hand sanitizer.

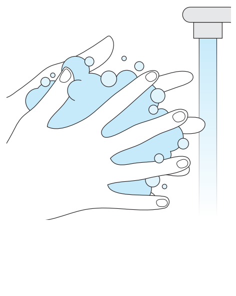

Please notify your prescribing Healthcare provider of the results of your QuickVue At-Home Covid-19 Test.

The QuickVue At-Home COVID-19 Test is for FDA Emergency Use Authorization (EUA) Only

For Prescription Use Only. For In Vitro Diagnostic (IVD) Use.

- This product has not been FDA cleared or approved but has been authorized by FDA under an EUA.
- This product has been authorized only for the detection of proteins from SARS-CoV-2, not for any other viruses or pathogens.
- The emergency use of this product is only authorized for the duration of the declaration that circumstances exist justifying the authorization of emergency use of IVDs for detection and/or diagnosis of COVID-19 under Section 564(b)(1) of the Federal Food, Drug and Cosmetic Act, 21 U.S.C. § 360bbb-3(b)(1), unless the declaration is terminated or authorization is revoked sooner.
- For more information on EUAs go here: *https://www.fda.gov/emergency-preparednessand-response/mcm-legal-regulatory-and-policy-framework/emergency-use-authorization*
- For the most up to date information on COVID-19, please visit: www.cdc.gov/COVID19
- For detailed instructions, please visit **www.quickvueathome.com**

Warnings, Precautions and Safety Information

- For prescription use only
- Read the written instructions fully before starting the test procedure
- To ensure correct results, you must follow the instructions
- Keep test kit and materials out of the reach of children and pets before and after use
- Wear safety mask or other face covering when collecting swabs from children or others
- Use of personal protection materials such as gloves are recommended
- Do not open the materials until ready for use. If the test strip is open for an hour or longer, invalid test results may occur.
- Improper swab collection may result in incorrectly negative (false negative) results
- The test is intended to be read at 10 minutes. If the test is read before this or is read more than 5 minutes after the indicated read time, results may be inaccurate and the test should be repeated.
- Do not use a test kit that is expired
- Do not touch the swab head when handling the swab
- Avoid exposure of your skin, eyes, nose, or mouth to the solution in the tube.

Hazardous Ingredients for Liquid Reagent

Chemical Name/CAS	Harms (GHS Code) for each ingredient	Concentration
Sodium Phosphate Monobasic Monohydrate/10049-21-5	Causes skin irritation (H315)<br>Causes serious eye irritation (H319)<br>May cause respiratory irritation (H335)	0.7%
Sodium Phosphate Dibasic Anhydrous/7558-79-4	Causes serious eye damage (H318)<br>Causes serious eye irritation (H319)	0.7%
C12-14-Alkyldimethyl-betaines/66455-29-6	Causes severe skin burns and eye damage (H314)<br>Causes serious eye damage (H318)<br>Causes skin irritation (H315)<br>Causes serious eye irritation (H319)	0.03%
ProClin® 300	Harmful if swallowed (H302)<br>Harmful if inhaled (H332)<br>Causes severe skin burns and eye damage (H314)<br>May cause an allergic skin reaction (H317)	0.03%
EDTA Tetrasodium Salt/64-02-8	Harmful if swallowed (H302)<br>Causes serious eye damage (H318)<br>Causes serious eye irritation (H319)<br>Harmful if inhaled (H332)<br>May cause respiratory irritation (H335)<br>May cause damage to organs (H371), single exposure	0.2%

- The solution in the tube contains hazardous ingredients (see table above). If the solution contacts the skin or eye, flush with plenty of water. If irritation persists, seek medical advice. https://www.poison.org/contact-us or 1-800-222-1222

Frequently Asked Questions

Will this test hurt?

No, the nasal swab is not sharp and it should not hurt. Sometimes the swab can feel slightly uncomfortable. If you feel pain, please stop the test and seek advice from a healthcare provider.

What are the known and potential risks and benefits of this test?

Potential risks include:

- Possible discomfort during sample collection.
- Possible incorrect test results (see Results section).

Potential benefits include:

- The results, along with other information, can help your healthcare provider make informed recommendations about your care.
- The results of this test may help limit the spread of COVID-19 to your family and others in your community.

You have the option to refuse this test. However, your doctor has prescribed this test because they believe it could help with your care.

What is the difference between an antigen and molecular test?

An antigen test, such as the QuickVue At-Home COVID-19 Test, detects proteins from the virus. Molecular tests detect genetic material from the virus. Antigen tests are very specific for the virus, but not as sensitive as molecular tests. This means that a positive result is highly accurate, but a negative result does not rule out infection. If your test result is negative, you should discuss with your healthcare provider on whether an additional test is necessary and if you should continue isolating at home.

How Accurate is this Test?

Based on the interim results of a clinical study where the QuickVue At-Home COVID-19 Test was compared to an FDA authorized molecular SARS-CoV-2 test, QuickVue At-Home COVID-19 Test correctly identified 84.8% of positive specimens and 99.1% of negative specimens.

Intended Use

The QuickVue At-Home COVID-19 Test is intended for the qualitative detection of the nucleocapsid protein antigen from SARS-CoV-2. This test is authorized for prescription home use with self-collected (unobserved) anterior nares (NS) swab specimens directly from individuals aged 14 years and older who are suspected of COVID-19 by their healthcare provider within the first six days of the onset of symptoms. This test is also authorized for prescription home use with adult-collected anterior NS samples directly from individuals aged 8 years or older who are suspected of COVID-19 by their healthcare provider within the first six days of the onset of symptoms.

The QuickVue At-Home COVID-19 Test does not differentiate between SARS-CoV and SARS-CoV-2.

Persons who test positive with the QuickVue At-Home COVID-19 Test should self-isolate and seek follow-up care with their physician or healthcare provider as additional testing may be necessary and for public health reporting.

Results are for the identification of SARS-CoV-2 nucleocapsid protein antigen. Antigen is generally detectable in anterior nares specimens during the acute phase of infection. Positive results indicate the presence of viral antigens, but clinical correlation with patient history and other diagnostic information is necessary to determine infection status. Positive results do not rule out bacterial infection or co-infection with other viruses and the agent detected may not be the definite cause of disease.

Negative results should be treated as presumptive and confirmation with a molecular assay, if necessary, for patient management may be performed. Negative results do not rule out COVID-19 and should not be used as the sole basis for treatment or patient management decisions, including infection control decisions. Negative results should be considered in the context of a patient's recent exposures, history and the presence of clinical signs and symptoms consistent with COVID-19. Persons who test negative and continue to experience COVID-19 like symptoms of fever, cough and/or shortness of breath may still have SARS-CoV-2 infection and should seek follow up care with their physician or healthcare provider.

All prescribing healthcare providers will report all test results they receive from individuals who use the authorized product to relevant public health authorities in accordance with local, state, and federal requirements using appropriate LOINC and SNOMED codes, as defined by the Laboratory In Vitro Diagnostics (LIVD) Test Code Mapping for SARS-CoV-2 Tests provided by CDC.

The QuickVue At-Home COVID-19 Test is intended for self-use and/or, as applicable for an adult lay user testing another person aged 8 years or older in a non-laboratory setting. The QuickVue At-Home COVID-19 Test is only for use under the Food and Drug Administration's Emergency Use Authorization.

Assistance

If the test does not perform as expected, call 833-QUICKVUE (833-784-2588) reference Say Yes! COVID Test.

Quidel Corporation
10165 McKellar Court, San Diego, CA 92121 USA
quidel.com

1488901 (03/21)
