# Supplementary material for: At-home testing to mitigate community transmission of SARS-CoV-2: protocol for a public health intervention with a nested prospective cohort study
Source: BMC Public Health. 2021 Dec 4;21:2209. doi: 10.1186/s12889-021-12007-w (PMC8642753; doi:10.1186/s12889-021-12007-w)
Supplement: Supplementary file 3 — Additional file 3. Summary of SYCT substudy activities. This table includes timing and content of questionnaires. [file 12889_2021_12007_MOESM3_ESM.docx]

**Additional file 3.** Summary of SYCT substudy activities including timing and content of questionnaires.

| **Study Period/Phase** | **Substudy Questionnaires/Surveys** | | | | | | | |
| --- | --- | --- | --- | --- | --- | --- | --- | --- |
|  |  | **Testing Phase** | | | | **Follow-Up Phase** | | |
| **Week** | **Baseline** | **1** | **2** | **3** | **4** | **5** | **13** | **21** |
| Contact Information | X |  |  |  |  |  |  |  |
| Socio-Demographic Data | X |  |  |  |  |  |  |  |
| Medical History | X |  |  |  |  |  |  |  |
| Anthropometrics – Weight | X |  |  |  |  | X |  |  |
| Anthropometrics – Height | X |  |  |  |  |  |  |  |
| Questionnaires |  |  |  |  |  |  |  |  |
| - Social interactions - Behaviors - Symptoms - Healthcare utilization | X | X |  | X |  | X | X | X |
| - Beliefs | X | X |  |  |  | X |  | X |
| - Knowledge of prevention approaches |  |  |  |  |  | X |  | X |
| - Feasibility of at-home testing |  |  |  |  |  | X |  |  |
| Testing Adherence/  Compliance reminders |  | X | X | X | X | X | X | X |
